# How Good Is Good Enough? Cookstove Replacement Scenarios to Reach Indoor Air Goals

**DOI:** 10.1289/ehp.123-A216

**Published:** 2015-08-01

**Authors:** Julie R. Barrett

**Affiliations:** Julia R. Barrett, MS, ELS, a Madison, WI–based science writer and editor, is a member of the National Association of Science Writers and the Board of Editors in the Life Sciences.

Air pollutants emitted by low-performing (i.e., high-polluting) cookstoves are estimated to cause 4 million premature deaths annually worldwide.^1^ High-performing cookstoves may mitigate health problems, but programs to disseminate these cleaner units have been plagued by incomplete adoption and often result in scenarios where both the old and new stoves are used simultaneously—so-called stove stacking. A new study in this issue of *EHP* examines just how much compliance is required in order to realize the benefits of cleaner technologies.^2^

The percentage of the global population using low-performing cookstoves dropped between 1980 and 2010, but absolute numbers have remained stable because of population growth.^3^ Approximately 3 billion people in the developing world prepare food over traditional cookstoves fueled by wood, coal, crop residues, or animal dung, which emit fine particulate matter (PM_2.5_), carbon monoxide (CO), and other pollutants during use.^3^ Exposure to these emissions contributes to the development of respiratory and heart diseases, low birth weight, and premature death.^4^ Traditional stoves are also estimated to produce 25% of the world’s output of black carbon, a potent climate forcer.^5^

**Figure:**
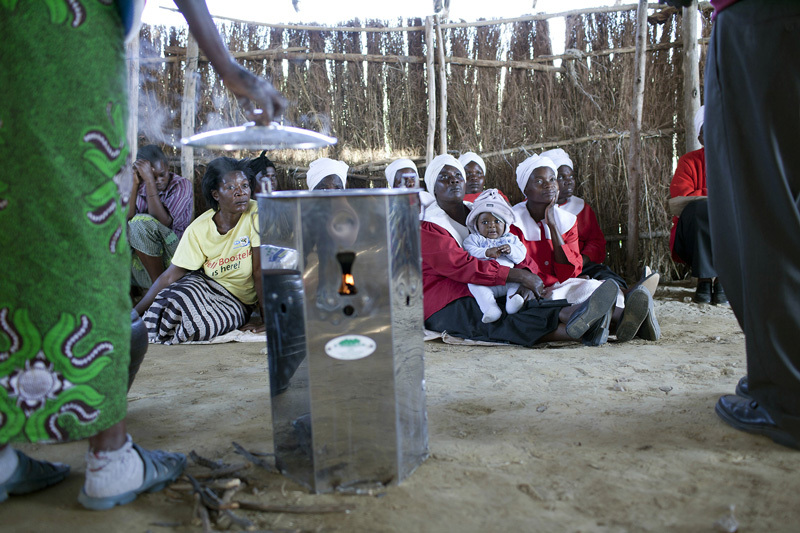
Zambian women receive a demonstration of a high-performing cookstove being distributed by the Clean Development Mechanism, which promotes reduction of greenhouse gas emissions in developing countries. © Per-Anders Pettersson/Getty Images

Despite the benefits of cleaner cookstoves, users may prefer older cooking technologies due to fuel availability, ease of use, and compatibility with local cooking demands—for instance, how well they work at preparing specific dishes.^6^ “There are lots of stories of where these alternate technologies work well for some tasks, but work really badly for others,” says Andrew Grieshop, an assistant professor in the Department of Civil, Construction, and Environmental Engineering at North Carolina State University, who was not involved in the study. “They’re not as universal as people would like them to be.”

To reap the benefits of cleaner cookstoves, though, not only is their sustained use required,^6^ they also need to displace low-performing units.^2^ “Ideally, of course, we’d love to have one hundred percent displacement with super-clean technologies, but we know it’s a transition that’s going to take quite a bit of time in some places,” says study coauthor Michael Johnson, a senior scientist at the Berkeley Air Monitoring Group.

The modeling framework presented by the authors includes predicted concentrations of PM_2.5_ and CO in kitchens based on the emissions performance of the stove(s) in use, ventilation room volume, and time spent cooking. The concentrations were predicted for scenarios ranging from 0% to 100% displacement of old stoves with new units, and a full day of cooking was assumed to be three 1-hour sessions.^2^

According to model estimates, a traditional three-stone fire—essentially, a pot balanced over an open fire on a trio of stones—could be used for only approximately 10 minutes per day before exceeding an interim PM_2.5_ limit of 35 µg/m^3^ set by the World Health Organization (WHO), whereas a traditional charcoal stove could be used for up to 25 minutes. Furthermore, a three-stone fire and a charcoal stove would reach CO limits within 75 minutes and 50 minutes, respectively. The only scenario that met WHO limits required the highest-performing cookstoves to nearly completely displace traditional stoves. Nevertheless, the model estimated that more modest reductions in use of traditional stoves could be expected to reduce the risk of adverse health outcomes.^2^

“[This model] is extremely useful in highlighting a key issue that I think the stove community is really only starting to grapple with now,” says Hisham Zerriffi, an assistant professor at the Liu Institute for Global Issues at the University of British Columbia. “This issue is less about the adoption of new stoves and more about the ‘disadoption’ of old stoves. What this study provides is further evidence of the need to think seriously about the factors that drive the way technologies get used in a household and which mix of technologies get used.” Zerriffi was not involved in the study.

The model is limited by factors such as uncertainty about real-world emissions, variability in ventilation, and assumptions about cooking times.^2^ “As a planning tool, however, it’s useful,” Grieshop says. “It makes the point that you have to be very aware that if you don’t completely replace the current technology, then you are not going to get the benefits—in some cases, you may not even get close.”

Johnson also notes that good field-testing is essential; the model and estimated impacts provide guidance, not absolutes. “Whatever groups ultimately choose to go forward with—in terms of technology, behavior change programs, finding financing mechanisms, and whatever will help implement their program—certainly verification on the ground is going to be critical to make sure that the intended impact is happening.”
